# Adjustment Disorder: epidemiology, diagnosis and treatment

**DOI:** 10.1186/1745-0179-5-15

**Published:** 2009-06-26

**Authors:** Mauro Giovanni Carta, Matteo Balestrieri, Andrea Murru, Maria Carolina Hardoy

**Affiliations:** 1 Centro per la Ricerca e la Terapia in Salute Mentale, Department of Public Health, University of Cagliari, Italy; 2 Clinica di Psichiatria e PMD, Dipartimento di Patologia e Medicina Sperimentale, University of Udine, Udine, Italy

## Abstract

**Background:**

Adjustment Disorder is a condition strongly tied to acute and chronic stress. Despite clinical suggestion of a large prevalence in the general population and the high frequency of its diagnosis in the clinical settings, there has been relatively little research reported and, consequently, very few hints about its treatments.

**Methods:**

the authors gathered old and current information on the epidemiology, clinical features, comorbidity, treatment and outcome of adjustment disorder by a systematic review of essays published on PUBMED.

**Results:**

After a first glance at its historical definition and its definition in the DSM and ICD systems, the problem of distinguishing AD from other mood and anxiety disorders, the difficulty in the definition of stress and the implied concept of 'vulnerability' are considered. Comorbidity of AD with other conditions, and outcome of AD are then analyzed. This review also highlights recent data about trends in the use of antidepressant drugs, evidence on their efficacy and the use of psychotherapies.

**Conclusion:**

AD is a very common diagnosis in clinical practice, but we still lack data about its rightful clinical entity. This may be caused by a difficulty in facing, with a purely descriptive methods, a "pathogenic label", based on a stressful event, for which a subjective impact has to be considered. We lack efficacy surveys concerning treatment. The use of psychotropic drugs such as antidepressants, in AD with anxious or depressed mood is not properly supported and should be avoided, while the usefulness of psychotherapies is more solidly supported by clinical evidence. To better determine the correct course of therapy, randomized-controlled trials, even for the combined use of drugs and psychotherapies, are needed vitally, especially for the resistant forms of AD.

## 

Learning Objectives: Upon the completion of this lecture the participants will be able to:

• understand problems and limits of a diagnosis based on 'response to stress'

• remember disorders usually found in comorbidity with AD and general outcome of the disorder

• know current treatment for AD

## Introduction

Stressful life events, even if brief, may influence ones health. These events may even lead to psychopathological alterations.

Diagnostic and Statistic Manual IV TR [1] basing on the importance of the causing effect, on symptoms reported and duration of the disorder, divides disorders which are strongly related to stressful life events into two main categories: Post-Traumatic Stress Disorder (PTSD) and Adjustment Disorder (AD). The former comes as a consequence of life-events such as life-threatening menaces, injury menaces or great physical or psychological distress. The latter, which will be later discussed, are also defined as "Adjustment Syndromes" [2] are conditions of subjective and emotional distress triggered as consequences of a meaningful change in life.

AD is commonly diagnosed by specialists, but it has found little place in the scientific literature: our aim is to clarify this condition in terms of diagnosis, aetiology and treatment by a critical review of the literature.

### Diagnosis of adjustment disorder

The concept of a wide range of symptoms following a psychosocial stressor has been present since DSM I [3]; the term 'adjustment disorder' first appeared in DSM III [4,5] and has evolved to the DSM IV definition. Despland et coll. in 1995 [6] substantially confirmed the validity of the AD diagnosis and pointed out that the course of a certain proportion of these disorders goes beyond the 6-month period stipulated by DSM-III-R [7]. This result supported the modifications introduced in DSM-IV [1].

In DSM IV [1], its essential feature is the development of clinically significant emotional or behavioral symptoms in response to an identifiable psychosocial stressor or stressors occurring within 3 month of the onset of the stressor (criterion A); these symptoms must be characterized by marked distress, in excess to what would be expected from exposure to the stressor, and significant impairment in social or occupational functioning.

The stress related disturbance does not meet the criteria for another Axis I disorder and must not be merely an exacerbation of a pre-existing Axis I or Axis II disorder.

Once the stressor has terminated, the symptoms may resolve within 6 months (Acute Adjustment disorder) or may persist for a longer period if the stressor has long term consequences (Chronic Adjustment Disorder). Bereavement is a diagnosis in DSM IV for those grief reactions that are abnormal. AD is not used in this instance.

DSM-IV TR criteria for the diagnosis of Adjustment Disorder are:

* Occurring within 3 months after the onset of a stressor.

* Marked by distress that is in excess of what would be expected, given the nature of the stressor, or by significant impairment in social or occupational functioning.

* Should not be diagnosed if the disturbance meets the criteria for another Axis I disorder or if it is an exacerbation of a pre-existing Axis I or II condition.

* Should not be made when the symptoms represent bereavement.

* The symptoms must resolve within 6 months of the termination of the stressor but may persist for a prolonged period (longer than 6 months) if they occur in response to a chronic stressor or to a stressor that has enduring consequences.

Different subtypes of adjustment disorder are listed in DSM IV:

With Depressed Mood (309.0), With Anxiety (3090.24), With Mixed Anxiety and Depressed Mood (309.28), With Disturbance Of Conduct (309.3), With Mixed Disturbance of Emotions and Conduct (309.4) and Unspecified (309.9)

The history of the adjustment disorder diagnosis in the official WHO classification is similar. ICD-10 [2], places adjustment disorder in a category of its own, separate from acute stress reactions and defines it as

* Occurring within 1 month of a psychosocial stressor that is not of an unusual or catastrophic type.

* The duration of symptoms does not usually exceed 6 months except for prolonged depressive reaction (in response to prolonged exposure to a stressful situation).

* The symptoms or behaviour disturbances are of a type found in any of the affective disorders but the criteria for an individual disorder are not fulfilled.

* Symptoms vary in severity and form.

WHO classification specifies that predisposition or individual vulnerability plays a greater role in conditioning the onset and symptoms of Adjustment Disorders than in other disorders of the same cluster (Neurotic Syndromes, F43), and disorder would not start without the stressor.

This implies a sort of "stress vulnerability syndrome", even if it does not correspond to a diagnostic group.

**Figure 1:**
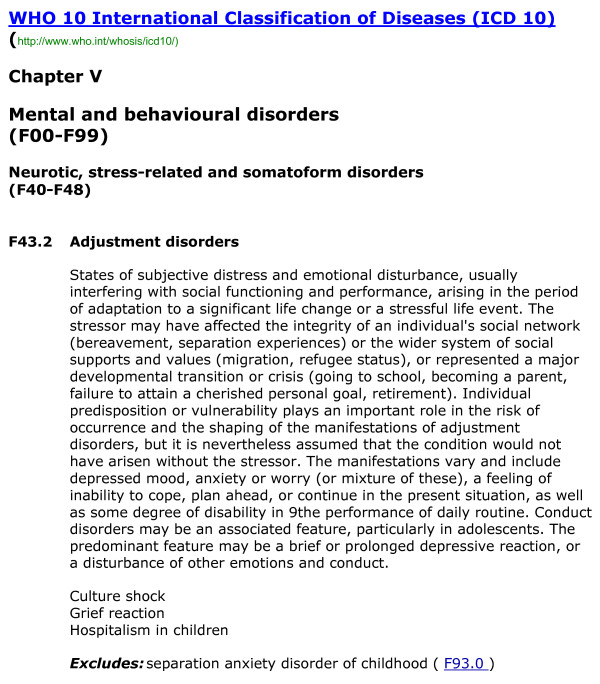
**WHO criteria**.

**Figure 2:**
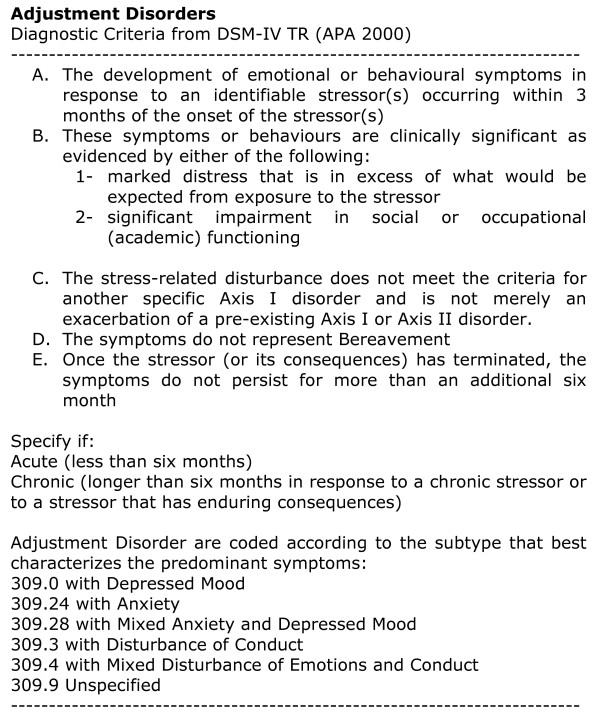
**APA criteria**.

Essentially, the core feature in the AD diagnosis (using either WHO [figure 1] or APA [figure 2] criteria) is clinically significant emotional or behavioral symptoms, often depressive in nature, that develop after an identifiable stressor [8]. The two main classifications differ in terms of the severity of impairment: ICD-10 points to "usually interfering with social functioning and performance" and "some degree of disability in the performance of daily routines" whereas DSM-IV points to "marked distress that is in excess of what would be expected given the nature of the stressor by significant impairment in social or occupational functioning" [9].

This type of diagnosis, in some ways, contradicts the principles that have guided modern psychiatric classifications. According to Strain and Diefelbacher [8], the DSM and ICD classifications were designed conceptually within an anti-theoretical framework to encourage psychiatric diagnoses to be derived on phenomenological grounds with an avowed dismissal of pathogenesis or etiology as diagnostic imperatives. In direct contradiction to this anti-theoretical approach, AD and the stress induced disorders require the inclusion of an etiologic significance to a stressor and the need to relate the stressor's effect on the patient in clinical terms. On a strictly descriptive level, the authors themselves underline that the diagnostic features of the AD, specifically: a) reaction to a significant stressor; b) mal-adaptation to the stressor with dysfunction in social and work activities; c) disturbance in mood, anxiety and conduct, are not given quantifiable criteria and this omission may obfuscate reliability and validity. They justify this lack of definitiveness, asserting that, "the lack of specificity allows the tagging of early or temporary mental states when the clinical picture is vague and indistinct, but the morbidity is greater than expected in a normal reaction"[10]

In this area of "problem-level diagnoses" – fairly loose sets of behavioural and social dysfunctions that are not considered disturbances in a strict sense – in contrast to those that require manifestation of a precise set of diagnostic criteria, we find the category of entities like AD where the symptoms correlate with an event, but with considerable variations. Such a category leads us into a set of ailments, perhaps "treatable with psychotherapy", that are conceptually in contrast to those "threshold – based diagnosis" ailments that, with their more rigid diagnostic criteria and their better-characterized pathophysiological targets that can be treated pharmacologically.

Indeed, if this hypothesis is true it could well help to explain why the diagnosis of AD has been eclipsed by the focus on mood disorders among researchers and policy markers [11]. Nevertheless, these concepts bring up some unresolved dilemmas. In the first place, it is not known if all "Sub-threshold" disturbances must, perforce, be triggered by stressful events. Consider for example Brief Recurrent Depression which is classified among the minor depressions but is not postulated to have any triggering event [12]. On a larger scale, even for the major depressions or anxiety disorders, it has never been shown, nor even hypothesized that a stressful event is always necessary for disease occurrence or evolution. Furthermore, it is known that persons who develop Social Phobia at a young age are at higher risk for successive anxious and depressive disturbances, and also show greater vulnerability to adverse reaction to stressful events, and this helps to appreciate just how complicated it will be to answer these questions [13].

Even those works that have put forth and validated diagnosis criteria for AD have not resolved the questions because patients with AD differ from those with no diagnosis and those with mood disorders on a number of parameters including differences in the nature of the stressors, outcome and quality of life [13]. Furthermore, the proposed criteria do not resolve if the parameters are merely related to the lesser gravity of symptoms of if they are related to specific differences between AD and depressive or anxious disorders [13].

Casey and Dowrick [13] affirm that there are two border disputes concerning the diagnosis of AD. One is the indistinct separation between the varied manifestations of AD from normal adaptive reactions. Casey [8] states that the conceptual problem lies in the following statement: 'the border between adjustment disorder and ordinary problems of life may be clarified by the notion that adjustment disorder implies that the severity of the disturbance is sufficient to justify clinical attention or treatment'. The application of the diagnosis based not just on objective criteria but on attempts to find a treatment opens up a deontological problem, and points out the limits of resolution in detecting psychiatric morbidity [6].

The second dispute is the problem of overlap with other disorders. Both ICD-10 and DSM-IV attempt to overcome this problem by specifying that if criteria for another disorder are met, then the diagnosis of AD should not be made; in essence the diagnosis is one of default. Given this, at present, most diagnoses of AD are essentially descriptive; it is not known if there are clear neurological or behavioural differences among patients in the course of developing, say Major Depressive Disorder (MDD), as distinct from AD, from those that suffer from AD [13]. Overall, the most that we can say of the current situation is that the efforts to identify hallmark differences between AD and more serious disorders have not yielded certain results.

Takei and Suguhara [14] pointed out the need to clarify the diagnostic borders in the subthreshold depression. Adjustment Disorder with Depressed Mood, in a dimensional diagnostic perspective, may be a "subsyndromal depressive disorder" and should be considered near the end of the depression spectrum [15].

Casey et al. [16], working on the ODIN study database, examined some variables that might distinguish AD from depressive episode and failed to identify any variables, even robust ones, such as BDI severity, that independently differentiated AD from depressive episode. Adjustment disorders may consist of either mild symptoms for a prolonged period or severe symptoms for a short period. In either case the condition needs careful evaluation and intervention as required.

At the moment, the distinction between AD and MDD can not be supported by biological data: Kumano et al. [17], as mentioned before, found that cancer patients who later developed MDD or AD showed regional brain metabolic changes. Though this study is interesting, it does not allow the distinction between those disorders. Furthermore, it does not permit an accurate prognosis between episodes that are self limiting and those that are not and that, therefore, require specific intervention.

Given this unclear situation, it is no surprise that the most common diagnostic tools may substantially be divided between those which pay no attention and those which pay little attention to Adjustment Disorder.

Many studies use as the Gold Standard a diagnosis derived from a clinical structured, or semi-structured interview using a tool like SCID [18]. As indicated above, given the complexities regarding diagnosis, it is not surprising that no questionnaire type instrument currently exists for AD diagnosis, although clinicians sometimes make a descriptive diagnosis using questions regarding the patients symptoms and their duration. Two other schedules, the Clinical Interview Schedule-Revised (CIS-R) [19] which was used in the British National Psychiatric Morbidity Survey [20] and the Composite International Diagnostic Interview [21] which was used in the U.S. National Comorbidity Study, [22] failed to incorporate AD in their assessments.

Again, Casey [8] notes that in Schedules for Clinical Assessment in Neuropsychiatry (SCAN) [23] the irrational disposal of the Adjustment Disorder items at the end of the interview in Section 13, dealing with Inferences and Attribution, after all other sections have been completed sends a clear message that this section is not as important as others. The effects of this on the diagnosis of adjustment disorder in epidemiological studies that use the SCAN would be an underestimation [8].

This may be true, but it seems a bit excessive to think that it may hold the reason of the surprising result in ODIN study which estimates Adjustment Disorder prevalence to be only 1%, with some sites failing to find any cases, despite the fact that the study was conducted in the general population in whom this condition is said to be common. The other possibility is that in the ODIN study, mild depression was conflated with AD [16]

Kirsh et al. [24], in a survey about prevalence of AD in a population of cancer patients undergoing bone marrow transplantation asserted that there is little accuracy in using existing scales for detecting adjustment disorders in cancer patients undergoing bone marrow transplantation, and that other tools for identifying patients with adjustment disorder who might benefit from counselling are needed. Later, Kirsh et al. [25] tried to assess the diagnosis of Adjustment Disorder by the use of a new tool, the C-Flex (Coping Flexibility Scale for Cancer), but he could not succeed in developing a specific scale. This may be because of problems with the scale or for the heterogenous nature of the AD category.

The difficulties in differentiating between AD and MDD are underscored in a study of Malt and colleagues [26] that examined the diagnostic reliability as part of the European Consultation Liaison Workgroup. The study design required that each consultant had to complete a training program for reliable use of the ICD/10 in Consultation-Liaison (C-L) psychiatry to be admitted to the reliability study. The partecipants were 220 psychiatrists and psychologists from 14 European countries. The training included rating of written test case vignettes and development of a coding manual to avoid diagnostic pitfalls not addressed in the ICD-10 manual. Following this training, all consultants rated 13 written case histories. 76% of consultants had a kappa of at least 0.70. Only 6% had a kappa of 0.40. But even at this high success rate, the consultants noted some problems in the differentiation between adjustment disorders and depressive disorders [26].

### Stress in adjustment disorder

In agreement with some points noted above, Fabrega et al. [5] underlined the debate if patients with AD have high personal vulnerability to common stressors or normal vulnerability to stronger stressors. Stressors causing AD may be of different types, and different weights. Classically, Paykel et al proposed a classification of life events by dividing them in desirable/undesirable (i.e.: career advancement/illness), entrance/escape(i.e.: wedding/death of a loved one) [27].

Selye [28] underlined the fact that some stressors may be easily won, and may even be positive (a low stress level, defined as "eustress", implies an increase in attention, concentration or memory). Selye then made a distinction between "eustress" and "distress" (defined as a negative stress).

Individual reaction to stressor may then be influenced by individual variables (age, gender, health level or psychiatric comorbidity), relative factors such as instruction level; ethical, political, religious beliefs; event/stressor being attended or not. Other variables may be found within the family environment: the presence or absence of affective support, relational strength, economic status.

Kumano [29] has gone beyond the obvious focus on the stressors observing there may be an individual vulnerability to the onset of psychiatric disorders: regional brain metabolic changes at 18-F-fluoro-deoxyg-lucose positron emission tomography (18-F-FDG PET) were present in cancer patients that later developed MDD or Adjustment Disorder; cancer patients who did not showed such changes, did not develop psychiatric disorders.

Brown [30-33] first proposed a stressor-vulnerability model to explain aetiology of depression. In his survey he observed that the occurrence of affective disorders had higher chance if certain kinds of life events and ongoing difficulties (provoking agents) combined with the presence of certain other social factors (vulnerability factors). Life stressors such as marked long-term difficulties and severe life events arising out of these difficulties. combine with individual response, 'negative' psychosocial factors (such as low self-esteem, inferred denial, self-blame and pessimism.) were found to be of particular importance in the development of depression. On the contrary, a 'positive' cognitive factor, that of downplaying, was inversely related to onset.

Brown and Harris [34] introduced the concept of subjectivity in stress evaluation, thus making efforts to measure stress in rating scales more complex: the same event may be very traumatic for one person and not be for another (i.e.: the death of a pet).

These results once again underline the difficulty in defining a reliable diagnostic for AD that is descriptive and reproducible. The process of diagnosis should consider the subjectivity of a stressor, and general or specific vulnerability to stressors (or that stressor in particular).

Even if the studies of Brown focused on depression, his findings on stress, vulnerability to it and its consequences in terms of a depressive reaction have clear implications for AD as well. In particular, the research into personal predisposition to a depressive reaction to stress and attachment style during childhood suggested that this may influence stress vulnerability [35] Mildly depressed individuals who reported a dismissing attachment style (higher levels of avoidant attachment and lower levels of anxious attachment) or preoccupied style (lower levels of avoidant attachment and higher levels of anxious attachment) experienced higher levels of stress associated with sociotropic events. Likewise, a dismissing attachment style predicted stress associated with dependent events among mildly depressed individuals. These effects were not present among more severely depressed participants to the study [35].

The studies concerning the relationship between psychopathology and vulnerability to life events reflects the fact that few studies were conducted in this field specifically concerning AD. At the present the questions, "Do people with AD have high vulnerability to common stressor or normal vulnerability to severe stressors?" and, "Are people with specific personality traits more prone to AD?" are still unresolved. Some studies may suggest future research lines. To this effect, recent papers have reignited debate concerning the relationship between stressful life events and depressive subtypes, particularly in relation to first versus subsequent episodes.

A study by Mitchell et al. [36] show that severe stressful life events (both acute and chronic) as defined by DSM-III-R axis IV-were more likely to occur prior to first rather than subsequent episodes, particularly for those with non-melancholic depression. These findings are consistent with other studies in suggesting an enhanced sensitisation of depressed patients to subsequent episodes of depression, but suggest that any such phenomenon is specific to non-melancholic depression. Others study are needed to confirm these results and investigate the relationship of AD with depressive subtype and if the presence of AD without sensitisation, in people coping with severe stress, may be a pattern characterizing these people versus those with high risk for depression.

A study of Ward and Colleagues [37] indicated that psychopathology diagnoses were associated significantly with mental representations of attachment classified in the Adult Attachment Interview (AAI), Preoccupied classification, according to AAI, was associated with Axis I diagnoses of affective disorders but the study did not analyze separately AD. However a study of Troisi et al., [38] studied the relationships between alexithymia, adult attachment style, and retrospective memories of separation anxiety symptoms during childhood in 100 young men with clinically significant mood symptoms in which the most common DSM-IV diagnosis (N = 72) was adjustment disorder with depressed mood, with anxiety, or with mixed anxiety and depressed mood. Each participant completed the Twenty-Item Toronto Alexithymia Scale (TAS-20), the Beck Depression Inventory (BDI), the state form of the State-Trait Anxiety Index (STAI), the Attachment Style Questionnaire (ASQ), the Relationship Questionnaire (RQ), and the Separation Anxiety Symptom Inventory (SASI). Alexithymic traits were more pronounced in those participants who had patterns of insecure attachment and who reported more severe symptoms of separation anxiety during childhood, independently of the severity of their current anxiety and depressive symptoms. Among the subgroup of participants with insecure attachment styles, those with preoccupied or fearful patterns had a higher prevalence of alexithymia (65% and 73%, respectively) than those with a dismissing pattern (36%). These data suggest a role for early developmental factors in the etiology of alexithymia. This study do not support the hypothesis that insecure attachment may increase the risk of AD (as suggested by the cited study of Bottonari [35]) but suggest that some determinants of outcome as alexitimia may be associated with insecure attachment, when AD occurred.

However a study of For-Wey and coll. in Taiwan [39] found statistically significant differences between cases (military personnel who met the DSM-IV criteria of AD) and an age-matched control group in personality and parental bonding attitudes. Soldiers with higher neuroticism, lower extroversion, and maternal overprotection had an increased risk of suffering from adjustment disorder. In accordance, a study investigating parenting received during childhood and early separation anxiety experiences in young male soldiers with adjustment disorder showed that compared with the controls, fifty-four conscripts suffering from adjustment disorder had significantly increased scores on the SCL-90-R, the Separation Anxiety Symptom Inventory (p < 0.03), and the father's and mother's Measurement of Parental Style Abuse subscale (p < 0.001) [40]. Finally, a patient's separation anxiety can be predicted from the mother's overcontrol behavior, and the severity of the disorder can be predicted from the father's abuse behavior [40]. These findings are in agreement with previous findings in patients with depression and anxiety disorders.

Early life stress, in particular child abuse and neglect, is an acknowledged risk factor for the development of pathology in adult life. Findings of a study of Vranceanu et coll. [41] support both direct and mediational effects of social resources on adult depression and PTSD symptoms in women with histories of child multi-type maltreatment, suggesting that resources are key factors in psychological adjustment of child multi-type maltreatment victims but the research didn't analyze the specific risk of AD versus MDD or PTSD.

### Epidemiology

Most of the large epidemiological surveys of the general population lack prevalence data for AD; this includes the Epidemiological Catchment Area (ECA) study [42], the U.S. National Comorbidity Survey (NCS) [43] and the U.K: National Psychiatric Morbidity Survey [20].

The only survey which included AD is the Outcome of Depression International Network (ODIN) [44] project. The objective of the ODIN project was to detect depressive disorders (including AD with depressed mood following ICD-X criteria), in rural and urban population, aged 18–64, in five European Countries. By using a two-step screening method, researchers quite surprisingly diagnosed AD in less than 1% of population affected by a depressive-like disorder. This may reinforce criticism of this diagnostic entity, but it has to be considered that one explanation for the low prevalence may be due to the limitations of the diagnostic tools used. ODIN used a depression rating scale: maybe the cut-offs were too high to detect AD. Additionally, the diagnostic was not tested against a gold standard for AD diagnosis and the measure of accuracy in detecting AD (as specificity, sensitivity and so) is unknown. As previously mentioned there is no clinical interview sufficiently robust in diagnosing AD so data produced with a screening test without any preliminary accuracy study against a diagnosis produced by a clinical structured or semi-structured interview are to be used very carefully.

Rundell JR [45] studied military personnel who were psychiatrically evaluated from the theater of operations during recent Operation Enduring Freedom (OEF) and Operation Iraqi Freedom (OIF). Over 80% of patients were evaluated during the first 6 months, and the most common diagnosis was adjustment disorders (37.6%), other Mood Disorders having a frequency of (22.1%). The lack of accurate tools for diagnosing AD, as Casey and other authors underlined [8,10,46], may have caused the prevalence of depressive disorders to be misinterpreted. Because of this manifest difficulty, and above all because often more serious mental health problems emerge in cohorts of veterans, the literature on veterans has many more works focusing on PTSD, substance abuse, pain and chronic fatigue syndrome.

Many works maintain that the Adjustment Disorder is an important pathology that is encountered commonly in psychiatry practice [47] but is most typically seen in primary care settings [8], and commonly used in liaison psychiatry, where it is purported to have an estimated incidence of 5–21% in psychiatric consultation services for adults [48]. Given this frequency, we will discuss AD in consultation-liason therapy in a separate section.

### Comorbidity

Comorbidity is not limited to personality disorder [49] but extends to other conditions such as substance abuse, especially in adults. Greenberg [50] found that, among those admitted with a diagnosis of adjustment disorder, 59% had a new primary diagnosis of substance use disorder at discharge and that, overall, 76% had either a primary or secondary diagnosis of substance abuse on discharge [45]. Comorbidity often leads to a poor outcome [51].

### Suicide risk

DSM-IV TR states that there is an increased risk of suicide and suicide attempts in patients with AD [1], but, given the two following considerations, suicide risk seems to be lower than in other Axis I disorders [52,53].

Research [54] demonstrates that the higher risk is for Major Depression (27%) while for AD it is only 4%; suicide attempts under alcohol abuse occurred more often among the AD group and the interval from the beginning of the disorder until the suicide attempt was significantly shorter within the AD group. In this group the attempts were not planned, in comparison with the MD group.

Greenberg [50] states that patients with Adjustment Disorder have a higher risk of suicide attempt instead, but confirms the former's assertion that suicidality in adjustment disorder is short-lived, thus the risk being not a barrier to early discharge and shorter admission.

Recent findings [52,44] suggest that the suicidal process (from first indications of suicidal ideation to completed suicide) is significantly shorter and rapidly evolving without any prior indications of emotional or behavioural problems in cases diagnosed with adjustment disorder compared to cases diagnosed with other disorders. This underlines the importance of assessing suicide risk in patients diagnosed with adjustment disorder

In contrast to the findigs for adults of male adolescent suicides using the method of the psychological autopsy states that AD diagnosis may be applied in about 25%of the cases [53]. As previously stated, chronicity and behavioral symptoms could be the strongest predictors of poor outcome [54], and, at 5 years follow up, 2% of patients diagnosed with Adjustment Disorder would have made a suicide attempt [54].

In a recent survey, Chiou [55] investigated the characteristics of adolescents who attempted suicide in Taiwan: of a 109 adolescent psychiatric inpatients sample retrospectively reviewed, 10% had a diagnosis of adjustment disorder, school stress (46%), parent-child conflict (25%) being the most common precipitating factors.

It was found that AD was the second most common psychiatric diagnosis among consequently referred non-psychotic outpatient adolescents [56], and 25% with AD had suicidal behaviour, of whome 9% had attempted suicide [57]. No difference was found in diagnostic co-morbidity between suicidal and non-suicidal AD patients. Compared with non suicidal AD patients, suicidal AD more often had suicide of a significant other as a precipitant stressor, previous psychiatric treatment, poor psychosocial functioning, dysphoric mood and psychomotor restlessness; male AD patients with suicidal behaviour were characterized by school related stressors, problem with the law and restlessness whereas female AD patients with suicidal behaviour were characterized by parental illness and internalized symptoms [56].

In conclusion the data are apparently somewhat contradictory; one hypothesis explaining this diversity may be that determinants of suicidal behaviour in AD are the same that influence the prise in charge (co-morbidity with personality disorders or substance abuse, parent-child conflict, school stress and so) thus probably the findings of research carried out on psychiatric clinical records show a high rate of suicide due to selection bias. Unfortunately, data from community surveys is lacking.

### Outcome

In the definition of Adjustment Disorder, there is an expectation of good outcome relative to symptoms remitting after the removal of the precipitating stressor.

In 1978, Looney [58] said that AD was found to be less severe and disabling than other major psychiatric disorders in terms of chronicity, length of hospitalization, and disposition.

A five year follow up study [59] demonstrated that 71% of patients diagnosed with adjustment disorder did not meet Research Diagnostic Criteria (RDC) criteria for any diagnosis, only 13% had a diagnosis of major depression and/or alcoholism, and 8% met the criteria for antisocial personality disorder. The validity of the AD category is only partially supported among adolescents: the adolescents' illnesses diagnosed after 5 years included schizophrenia, schizoaffective disorder, major depression, bipolar disorder, antisocial personality, alcoholism, and drug use disorder. Chronicity and behavioral symptoms were the strongest predictors of poor outcome. A 5-year follow-up study [60] of 76 patients from a crisis intervention ward who were given an ICD-9 diagnosis of adjustment disorder confirmed the good prognosis associated with this condition: only 17% had developed a chronic or severe course and 2% committed suicide.

Studying consecutive patients in a hospital emergency department during the first 6 months after a serious accident, Kuhn et al. [61] found an incidence of Adjustment Disorder (1.5%) and that also the incidence of Acute Stress Disorder and subsyndromal Acute Stress Disorder were increased as a reaction to the accident. Six-months after the accident, 10% of the subjects met criteria for Major Depression, 6% for PTSD, 4% for subsyndromal PTSD, and 1.5% for Specific Phobia as newly developed disorders. Those patients who met criteria for any psychiatric diagnosis shortly after the accident (29% of a 58 patient sample) ran a much higher risk of developing new or comorbid psychiatric disorders in the future.

Weidenhammer [62] studied a total of 171 patients presenting with chronic fatigue who fell into three diagnostic categories, i.e. neurasthenia, affective disorders, adjustment disorders, before treatment. Treatment success was higher in the adjustment disorder and affective disorder groups.

Greenberg [50] studied subtyping, demographic variables, suicidal tendency, diagnostic stability, and 2-year rehospitalization outcome for inpatients given the admission diagnosis of adjustment disorder at their institution. He confirmed that adolescents and adults with adjustment disorder had a significantly shorter index of hospitalizations and more presented suicidality than the comparison subjects. Adults – but not adolescents – with adjustment disorder had significantly fewer psychiatric readmissions and fewer rehospitalization days 2 years after discharge than comparison subjects, and more adults with adjustment disorder had diagnoses of comorbid substance use disorder. A more careful observation during hospitalization caused about forty percent of the patients admitted with the diagnosis of adjustment disorder being discharged with different diagnoses. Only 18% of the inpatients with adjustment disorder who were rehospitalized were diagnosed as such at readmission.

In a Danish survey [63] the role of psychotropic drugs seems to be of negative impact on the outcome of the occupational rehabilitation of patients with stress-related adjustment disorders: the use of such drugs is the only negative predictor, whereas somatic drug treatment, age, gender, skill, workplace, matrimony, and smoking all were without any significant influence on work ability.

### Treatment

The fact that adjustment disorders are short-lived and resolve with the passage of time may explain the paucity of studies on the therapy of the disorder especially randomized controlled trials, but no longer justify the idea that no specific intervention is required, unless the individual is acutely suicidal. Clearly, patients with AD, are important subjects for research into prevention, and those who have more prolonged AD are also deserving of this same scientific concern due to decrease of the quality of life [8].

It is a shared opinion that currently, psychotherapy remains the treatment of choice for adjustment disorders [64], and we lack major pharmacotherapy studies to support antidepressant treatment. Unfortunately, psychotherapy is not very accessible: AD is often diagnosed in general practice.

The problem of which psychotherapy may be useful in adjustment disorders cannot find a certain answer, due to lack of controlled clinical trials of different psychotherapies.

The very definition of the disorder (a short-term difficulty, related to a stressor, that rarely goes beyond 6 months) suggests a solution-focused therapy, that help the individual deal more effectively with the specific life problem, like interpersonal psychotherapy or problem solving therapy [65]

A study on adolescents with major depression or other Depressive Disorder (among them Adjustment Disorder) [66] showed that psychosocial functioning improved in all whether their treatment involved only psychotherapeutic treatments or additional psychotropic medication.

In patients with AD brief psychotherapies can be useful according to Sifneos [67]. Unfortunately data on efficacy of brief psychotherapies in AD are scarce.

Maina et al. [68] in 1999 pointed out the effectiveness of brief dynamic psychotherapy and brief supportive psychotherapy in the treatment of minor depressive episodes, and the superior improvement in a 6 months follow up of the dynamic approach. Unfortunately the trial did not study the efficacy of brief dynamic psychotherapy in AD but this study may suggest the focus of future studies due to possible overlap of the two diagnoses.

Interpersonal psychotherapy was found to be effective in human immunodeficiency virus (HIV)-positive inpatients with depressive symptoms. In a randomized 16-week clinical trial comparing interventions with interpersonal psychotherapy, cognitive behavioral therapy, supportive psychotherapy, and supportive psychotherapy with imipramine for HIV patients with depressive symptoms; subjects randomized to interpersonal psychotherapy and supportive psychotherapy with imipramine had significantly greater improvement on depressive measures than those receiving supportive psychotherapy or cognitive behavioral therapy [69]. Interpersonal approaches include psychoeducation about the patient's role, a here and now frame work, formulation of the problems fron an interpersonal perspective, exploration of options for changing dysfunctional behavior pattern [70].

In 2005, Jojic [71,72] underlined the usefulness of autogenic training in a sample of adolescent patients, and later that year on adults too, with diagnosis of adjustment disorder: autogenic training significantly decreases the levels of physiological indicators of adjustment disorder (blood pressure, pulse rate, concentration of cholesterol and cortisol), diminishes the effects of stress in an individual, thus helping patients to cope with stress, and facilitates their recuperation.

Frankel [73] suggested the utility of the "ego-enhancing-therapy" for the treatment of AD in the elderly. This approach promotes the coping strategy and the and helps the patient acknowledge the stressors.

A study by Gonzales-Jaimes and Turnbull-Plaza [74] compared three different treatment types against AD in patients with acute myocardial infarction (AMI). Authors used a quasi-experimental design for four groups with three evaluations each, and conducted a random test of 144 patients of both genders between 30 and 60 years of age diagnosed with AMI and AD. Treatment evaluation was carried out with depressive description test (DDT) and MMPI-2 test, and depression (DEP) and health concerns (HEA) content scales. In post-test evaluation the authors found a significant difference between patients in the psychotherapy mirror group and the remaining groups (Gestalt psychoterapy, medical conversation and a control group without emotional support). During the 6-month follow-up evaluation, they observed a significant difference between the control group and the remaining groups on the HEA content scale. Patients with AMI and AD not receiving emotional support treatment in conjunction with medical treatment continued to experience emotional disorders and show greater apprehension with regard to medical treatments. Mirror therapy include psychosocial, cognitive and neurolinguistic components, the focus being to encourage the patient acceptance of psysical condition.

The only Randomized Controlled Trial found in literature about efficacy of Psychoterapy in AD was the study of Van der Klink ad coll. [75] that compared the "activating intervention" with "care as usual" (control group) for the guidance of employees on sickness leave because of an adjustment disorder. It was hypothesised that the intervention would be more effective than care as usual in lowering the intensity of symptoms, increasing psychological resources, and decreasing sickness leave duration. Symptom intensity, sickness duration, and return to work rates were measured at 3 months and 12 months. Analyses were performed on an intention to treat basis: at 3 months, significantly more patients in the intervention group had returned to work compared with the control group. At 12 months all patients had returned to work, but sickness leave was shorter in the intervention group than in the control group. The recurrence rate was also lower in the intervention group. There were no differences between the two study groups with regard to the decrease of symptoms. At baseline, symptom intensity was higher in the patients than in a normal reference population, but decreased over time in a similar manner in both groups to approximately normal levels. They concluded that the experimental intervention for adjustment disorders was successful in shortening sick leave duration, mainly by decreasing long term problems

The "activating intervention" was based on a three stage model, resembling stress inoculation training, a highly effective cognitive behavioural approach. In the first stage, there was emphasis on information: understanding the origin and cause of the loss of control. Patients were also stimulated to do more non-demanding daily activities. In the second stage, patients were asked to draw up an inventory of stressors and to develop problem solving strategies for these causes of stress. In the third stage, patients put these problem solving strategies into practice and extended their activities to include more demanding ones. The patients' own responsibility and active role in the recovery process was emphasised [75]. This study was the basis of the "Dutch practice guidelines for managing adjustment disorders in occupational and primary health care" [76].

Drug therapy may be a useful tool in treating Adjustment Disorder.

De Leo [77] claims that psychotherapy and drug therapy produced a significant improvement in a 4 week trial, divided in five groups: supportive psychotherapy (psychoanalytically oriented), viloxazine (an antidepressant), lormetazepam (a benzodiazepine), and S-adenosylmethionine (a methyl donor with antidepressive properties) and a placebo group. However, groups given S-adenosylmethionine and supportive psychotherapy had the highest mean scores.

A study of pattern prescription of antidepressant drugs in the nineties [78] showed significant increase in the prescription by office-based psychiatrists, greatest for patients with less severe psychiatric disorders (among them, AD). This suggested that in the common practice AD is considered from a symptomatic point of view as a depression, with no attention to the concept of time-limited and stress related event that truly defines it.

In a retrospective analysis which aimed to distinguish different response to antidepressant therapies between Major Depression and subsyndromal depressions (adjustment disorder with depressed mood between them) patients with adjustment disorder demonstrated no difference in clinical response to any particular antidepressant. The main statistical difference was in response rates, where patients diagnosed with adjustment disorder were twice as likely to respond to standard antidepressant treatment (approximately 70% of the time) than depressed patients. Neither single antidepressant was found to be more effective than another agent in treating adjustment disorder, nor combining antidepressants improved symptom relief over mono-therapy at four months. [58].

A study [79] comparing efficacy and safety of trazodone versus clorazepate in HIV-positive subjects with AD showed a similar profile in successfully treating the disorder, but with no risk of abuse and dependence.

Nguyen [80] in 2006 explored the differences in treating Adjustment Disorder with Anxiety with etifoxine (a non-benzodiazepine anxiolytic drug) and lorazepam: the results were that both drugs demonstrated efficacy in the treatment of the disorder, but more etifoxine patients improved markedly and had a notable therapeutic effects without side effects. Moreover, 1 week after stopping treatment, fewer etifoxine patients experienced a rebound of anxiety, compared to the others.

Bourin [81] suggested the superiority of plant extracts (Euphytose) versus placebo in the treatment of adjustment disorder with anxious mood.

In a randomized, double-blind placebo-controlled trial, Woelk [82] pointed out the effectiveness of Ginkgo Biloba in a sample of 107 patients with generalized anxiety disorder (n = 82) and adjustment disorder (n = 25). Changes were significantly different from placebo for both high-dose group and low-dose group, with a dose-response trend.

In a study of Volz and coworkers, [83] outpatients suffering from anxiety of non-psychotic origin (DSM-III-R criteria: agoraphobia, specific phobia, generalized anxiety disorder, and adjustment disorder with anxiety) were included in a 25-week multicenter randomized placebo-controlled double-blind trial with WS 1490, a special extract of kava-kava. There was a significant superiority of the test drug starting from week 8 in reducing hanxiety symptoms (measued by Hamilton Anxiety Rating Scale ans Self-Report Symptom Inventory-90 Items revised) and reducing the score at Clinical Global Impression Scale Adverse events were rare and distributed evenly in both groups. These results support WS 1490 as a treatment alternative to tricyclic antidepressants and benzodiazepines in anxiety disorders, with proven long-term efficacy and none of the tolerance problems associated with tricyclics and benzodiazepines. Nevertheless, Kava extracts are not available in many countries, and from 1990 to 2002, 83 cases of adverse liver reaction were reported, principally in Germany. In the face of the initial reports of toxicity, many European countries banned the sale of Kava extract.

### AD in consultation-lliaison (CL) psychiatry

The problems pointed out in the section on diagnosis re-emerge in CL psychiatry. But not only has the lack of a clear definition prevented AD study in CL psychiatry. Probably the main factor is the advent of easily managed antidepressants that render the psychiatrist more prone to use the MDD diagnosis and treat as such. In fact, an observational study of referrals to CL psychiatric units conducted in America over the course of 10 years found that from 1988 and 1997 the percentage of diagnosis of major depression in patient with concomitant medical illness increased from 6.4% to 14.7%. In the same period Ad with depressed mood decreased from 28% to 14.7% [84].

Still there are other problems as well, i.e. considering particularly stressing situations like patients with a painful serious illness or an illness with serious impairment. Bakr [85] recently studied the frequency of adjustment disorder and other psychiatric disorders in a sample of 19 children with predialysis chronic renal failure and 19 children with end-stage renal disease on regular hemodialysis. Adjustment disorder was the most common diagnosis (18.4%), followed by depression (10.3%) and neurocognitive disorders (7.7%). The disorders were more prevalent in dialysis (68.4%) than in predialysis patients (36.8%).

Mehnert [86] has studied acute and post-traumatic responses to stress, and comorbid mental disorders in breast cancer patients, revealing that, in a 127-post-surgery patients sample, Adjustment Disorder was the most common diagnosis (7.1%), followed by Generalized Anxiety Disorder (6.3%) and MDD (4.7%). In contrast, Ronson [87] expressed the opinion that in the context of the emotional response of cancer the diagnosis of AD risks to be inappropriate, because of the difficulty to define the level of what represents an "excessive" response. Consequently, it would be better to consider a subthreshold depression or a full or partial presentation of post-traumatic stress disorder (PTSD). In fact, according to the review of Ronson [86] an average of 10% of cancer patients has been shown to meet criteria for PTSD.

Notwithstanding these assertions, the diagnosis continues to be used in CL psychiatry. In the early 1990s, two studies carried out in the general hospital population found that the AD diagnosis was assigned in 21% and in 11% of psychiatric referrals [88,89].

A few years later, a multi-site study reported that a diagnosis of AD was made in 12% of psychiatric consultations [47]. More specifically, AD was the sole diagnosis in 7.8%, while it was assigned in comorbidity with other DSM-IV Axis I and II diagnoses in 4.2%. AD with depressed mood, anxious mood, or mixed emotions were the commonest subcategories used, while the most frequent comorbid diagnoses were personality disorders and organic mental disorders. As compared with patients with other psychiatric diagnoses, patients with AD were referred significantly more often for problems of anxiety, coping and depression. Moreover, they had less past psychiatric illness, were rated as functioning better and were more often found to have a current neoplasm. Notwithstanding these differences, interventions were similar to those for other Axis I and II diagnoses, in particular the prescription of antidepressants.

The latter finding poses the question of the treatment of AD in the clinical setting. In a recent critical paper, O'Keeffe et al. [90] argued that a diagnosis of depression runs the risk of the medical labelling of normal emotional reactions and offers unnecessary treatments, usually psychopharmacological. On the other hand, they acknowledged that the downside of using a lesser known diagnostic term, such as AD, is that it often results in confusion among physicians and patients. A further risk underlined by these Authors is that the use of the diagnosis of AD to describe clinical reactions considered transient or understandable may lead to adopt psychological therapies for this diagnosis, while antidepressants are limited to clinical depression. To contrast this risk, they concluded that the clinician should avoid disputing diagnostic labels, while, in the spirit of the biopsychosocial model, they should be able to use in the same patient antidepressants for anhedonia, psycho-educational or cognitive approaches to deal with maladaptive adjustment and behavioural activation for poor motivation and learned helplessness.

In summary, in the CL psychiatry practice the AD continues to be a frequent diagnosis, which despite the apparent lesser severity as compared to clinical depression represents a burdening and time-consuming disorder. The main problems of this diagnosis are its instability – as noted above, Greenberg et al. [49] found that a substantial number of patients admitted to a hospital with a diagnosis of AD were discharged with a different diagnosis – its vague boundaries with depression and PTSD risks leading the clinicians to consider it the emotional response as an inevitable consequence of the illness, or on the contrary to start unnecessary drug treatments.

## Conclusion

AD is a very common diagnosis in clinical practice, but we still lack data about its rightful clinical entity. This may be caused by a difficulty in facing, with purely descriptive methods, a "pathogenic label", based on a stressful event, to which a subjective impact has to be considered.

We lack efficacy surveys concerning treatment.

The use of psychotropic drugs such as antidepressants, in AD with anxious or depressed mood is not properly founded and should be avoided in less severe forms of this disorder.

More solid evidence has been produced about the usefulness of psychotherapies.

Data from randomized-controlled trials would be particularly interesting, also in resistant forms, even with combined use of drugs and psychotherapies.

## Competing interests

The authors declare that they have no competing interests.

## Authors' contributions

MGC participated in the manuscript revision, assessing the overall structure of the study and wrote the consultation psychiatry section. MB participated in the bibliographic research, in the manuscript revision, figures creation and manuscript editing. AM participated in the bibliographic research, articles selection and manuscript editing. MCH participated in the articles selection, manuscript editing and in the assessment of the overall structure of the study.
